# MiR-187 overexpression inhibits cervical cancer progression by targeting HPV16 E6

**DOI:** 10.18632/oncotarget.17516

**Published:** 2017-04-29

**Authors:** Mao Lin, Xiang-Yang Xue, Shu-Zhen Liang, Yin-Xiong Li, You-Yong Lv, Li-Hua He, Ke-Cheng Xu, Li-Fang Zhang, Ji-Bing Chen, Li-Zhi Niu

**Affiliations:** ^1^ Fuda Cancer Hospital, Jinan University School of Medicine, Department of Central Laboratory, Guangzhou, China; ^2^ Wenzhou Medical University, Department of Immunology and Microbiology, Wenzhou, China; ^3^ Guangzhou Institute of Biomedicine and Health, Chinese Academy of Sciences, Guangzhou, China; ^4^ Peking University Cancer Hospital, Department of Central Laboratory, Beijing, China

**Keywords:** microRNA-187, HPV16 E6, cervical cancer, occurrence, progression

## Abstract

Aberrantly expressed microRNAs contribute to the initiation and progression of human cancer. MiRNA-187 has been reported in nasopharyngeal, renal, pancreatic, prostate, and esophageal cancer, and acts as a tumor suppressor or oncogene. However, the underlying function of miRNA-187 in cervical cancer remains largely unexplored. In the present study, we demonstrated significantly miRNA-187 down-regulation in cervical cancer tissues and cell lines compared to their normal counterparts. Kaplan-Meier analysis revealed that decreased miRNA-187 was closely associated with shorter overall survival and relapse-free survival. Gain- and loss-of-function studies showed that miRNA-187 suppressed cervical cancer cell proliferation, migration, and invasion, and promoted cervical cancer cell apoptosis. Furthermore, luciferase reporter assay determined that human papillomavirus 16 E6 was a direct functional target of miRNA-187. Taken together, our findings indicate the essential role of miRNA-187 in suppressing cervical cancer progression and indicate a novel link between miRNA-187 and human papillomavirus 16 E6 in cervical cancer.

## INTRODUCTION

After breast and colorectal cancer, cervical cancer (CC) is the third most common cancer in women, causing an estimated 530,000 new cases and 275,000 deaths per year [1–3]. Due to the lack of screening and early diagnosis, about 85% of new cases occur in lower socioeconomic areas [4, 5]. Persistent human papillomavirus (HPV) infection is widely recognized as a cause of both cervical intraepithelial neoplasia (CIN) and cervical cancer. Current therapeutics, including surgery, radiation, and chemotherapy, show limited effectiveness for advanced invasive CC [1]. CC remains the most common cause of cancer-related death in women from developing countries. Identifying novel prognostic predictors or therapeutic targets may improve prognosis.

MicroRNAs (miRNAs) are a class of small (approximately 20–22 nucleotides), endogenous noncoding RNA molecules [6]. miRNAs mediate negative post-transcriptional regulation by base pairing with the 3′ untranslated regions (3′ UTRs) of one or more target genes [7]. Accumulating evidence has demonstrated that aberrantly expressed miRNAs act as tumor suppressors or oncogenes in human cancer, including colorectal cancer, breast cancer, lung cancer, and prostate cancer [8–10]; deregulated miRNA expression could be used as biomarkers of cancer risk, diagnosis, and prognostic prediction, and such miRNAs may even be potential therapeutic targets [11, 12]. Recent studies have shown significant miR-187 upregulation in many human malignancies. For example, high miR-187 expression in breast cancer leads to a more aggressive, invasive phenotype and acts as an independent predictor of outcome [13]. In ovarian cancer, excessive miR-187 promotes tumor progression through by disabled homolog 2 (DAB2), inhibiting epithelial–mesenchymal transition [14]. But in pancreatic cancer, low miR-187 expression predicts short overall survival (OS) in patients after radical surgery [15]. However, its possible functions and underlying mechanisms in CC have not been reported.

In the present study, we report significantly decreased miR-187 in CC tissues and cell lines. MiR-187 overexpression suppressed CC cell proliferation, migration, and invasion, and promoted CC cell apoptosis. Conversely, miR-187 knockdown significantly facilitated the malignant phenotype of CC cells. Furthermore, we identified HPV16 E6 as a direct and functional target of miR-187. Therefore, our results suggest that miR-187 may play crucial roles in CC development and progression by targeting HPV16 E6.

## RESULTS

### Specificity analysis of miR-187

**Figure 1:**
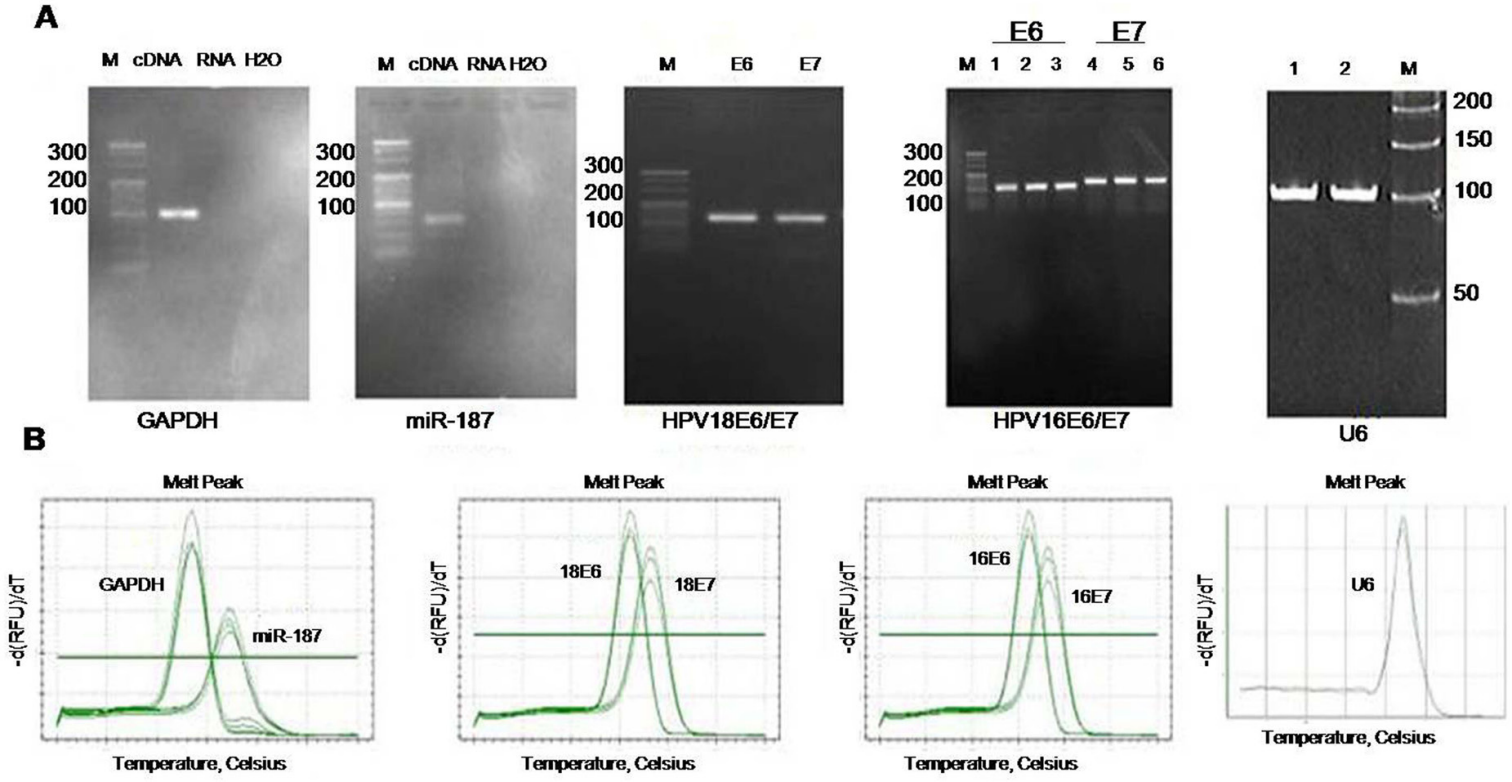
Specific analysis of each PCR primer **(A)** Image shows 4% agarose electrophoresis of GAPDH, miR-187, HPV18 E6/E7, HPV16 E6/E7, and U6 PCR products. **(B)** Melting curves of GAPDH, miR-187, HPV18 E6/E7, HPV16 E6/E7, and U6 PCR products. M: 20-bp DNA marker.

The melting curves of the quantitative PCR (qPCR) amplification products were all unimodal (Figure 1A), and the amplification products from total tissue RNA were all visualized as single bands following reverse transcription–qPCR (RT-qPCR) using specific primers (Figure 1B). These findings indicated that established RT-qPCR methods can detect the corresponding target genes of miR-187.

### MiR-187 is downregulated in CC and correlates with CC prognosis

**Figure 2:**
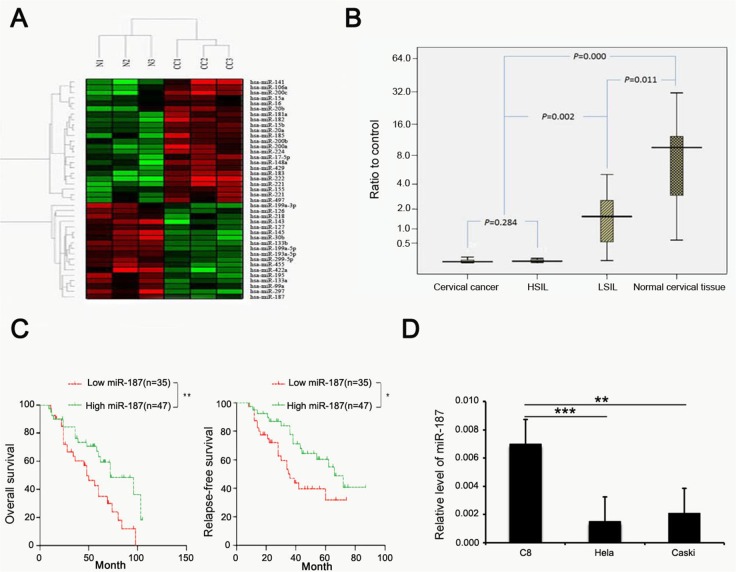
miR-187 expression is low in CC and correlates with prognosis of CC **(A)** Heatmap analysis of the expression of 41 miRNAs in normal cervical tissues and CC tissues, miR-187 expression is low. **(B)** Compared with normal cervical tissue, miR-187 is significantly decreased in CC specimens and cervical lesion tissues (*P < 0.05) and is decreased in CC specimens and HSIL relative to LSIL (*P < 0.05). **(C)** Kaplan–Meier curves for patients grouped based on miR-187 expression (*P < 0.05; **P < 0.01). **(D)** Histograms of miR-187 transcription levels in HeLa and CaSki cells and in the normal C8 cells (**P < 0.01; ***P < 0.001).

Figure 2A showed the heatmap analysis of the expression of 41 miRNAs in normal cervical tissues and CC tissues obtained from microarray data. Twenty-three miRNAs were over-expressed and eighteen miRNAs, including miR-187, were low expression.

To elucidate the pattern of miR-187 expression in CC, we first detected miR-187 expression levels in 209 cervical tissue samples using real-time PCR, which included 82 carcinoma tissues and the matched normal tissues (5 cm distal from the tumor), and 45 cervical squamous intraepithelial lesions. MiR-187 was significantly decreased in the CC specimens and cervical squamous intraepithelial lesions relative to the normal control, and was decreased in CC specimens and high-grade squamous intraepithelial lesion (HSIL) tissues compared to low-grade squamous intraepithelial lesion (LSIL) tissue (Figure 2B). We also analyzed the prognostic value of miR-187 expression in patients with CC. Kaplan–Meier curves revealed higher rates of OS and relapse-free survival of the high miR-187 expression group than in the low miR-187 expression group (Figure 2C).

Subsequently, we measured miR-187 expression levels in two CC cell lines (HeLa cells and CaSki cells) and in the normal C8 cervical epithelial cell line using real-time RT-PCR (Figure 2D). MiR-187 expression was lower in the CC cells (HeLa cells, HPV18-positive; CaSki cells, HPV16-positive) as compared to the C8 cells. Collectively, these findings indicated that low miR-187 expression correlates with the clinical outcome in CC and might play a role in CC development or progression.

### Association between miR-187 expression and the clinicopathological parameters of CC

miR-187 expression and age and pathological type were not significantly correlated. There was a significant difference between miR-187 expression and pathological grade, clinical stage, tumor size, and lymph node metastasis (Table 1). Low miR-187 expression was significantly associated with pathological grade, clinical stage, nodal metastasis, and larger tumor size (all, P *<* 0.05), suggesting that decreased miR-187 expression may be involved in CC progression and metastasis.

**Table 1:** Association between miR-187 expression and clinicopathological parameters of CC

Clinicopathological parameters	Total (n = 82)	miR-187 relative expression	P
**Age, years**			
<35	13	0.701 (0.372, 0.798)	0.903
≥35	69	0.600 (0.275, 0.830)	
**Pathological grade**			
1	11	0.980 (0.800, 1.360)	0.017
2	52	0.650 (0.360, 0.740)	
3	19	0.350 (0.220, 0.780)	
**Tumor size, cm**			
<4	21	0.270 (0.200, 0.800)	0.005
≥4	61	0.890 (0.640, 0.900)	
**Lymph node metastasis**			
Negative	24	0.690 (0.570, 0.820)	0.04
Positive	58	0.290 (0.210, 0.820)	
**Clinical stage**			
I/II	19	0.770 (0.630, 1.140)	0.026
III/IV	63	0.370 (0.190, 0.790)	
**Pathological type**			
Squamous carcinoma	70	0.390 (0.180, 1.360)	0.119
Adenocarcinoma	12	0.750 (0.510, 0.940)	

### The best miR-187 intervention time and concentration

**Figure 3:**
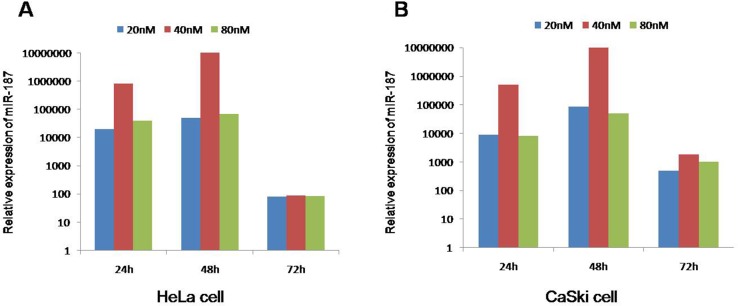
The best intervention time is 48 h and 40 nM of miR-187 mimics due to the maximum level of miR-187 Intervention times (24 h, 48 h, 72 h) and intervention concentrations (20 nM, 40 nM, 80 nM) of miR-187 in **(A)** HeLa cells and **(B)** CaSki cells.

To better elucidate the effect of miR-187, we treated HeLa cells and CaSki cells for 24 h, 48 h, and 72 h with 20 nM, 40 nM, and 80 nM miR-187. The best miR-187 treatment duration and concentration was 48 h and 40 nM, respectively (Figure 3A, 3B), where miR-187 levels were highest in the cultures.

### Deregulated miR-187 expression regulates HPV16 E6 mRNA and p53 protein expression

**Figure 4:**
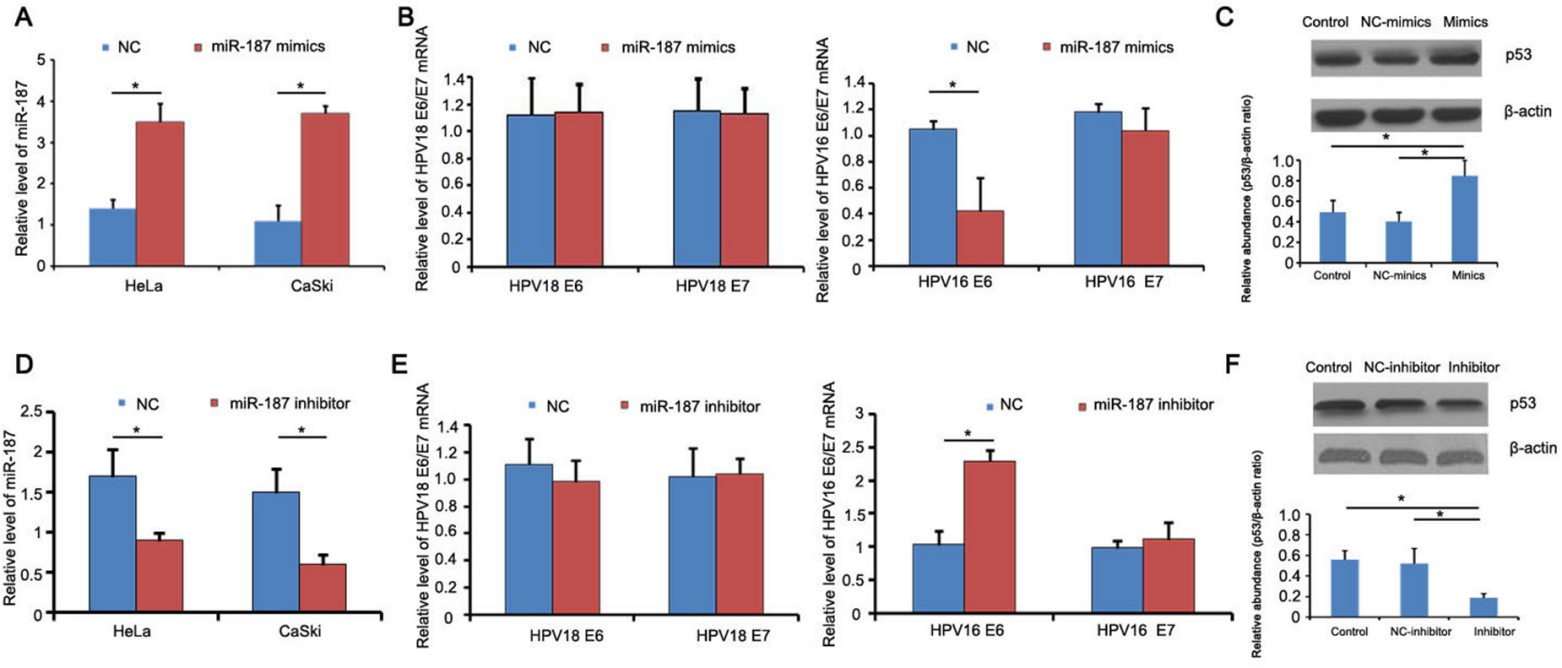
miR-187 affects the expression of HPV16 E6 mRNA and p53 protein **(A)** Detection of miR-187 expression levels after mimics transfection. **(B)** Relative expression of HPV18 E6/E7 and HPV16 E6/E7 mRNA; HPV16 E6 mRNA was decreased (*P < 0.05). **(C)** Corresponding increased expression of p53 protein compared to NC and blank groups (*P < 0.05). **(D)** Detection of miR-187 expression levels after inhibitor transfection. **(E)** Relative expression of HPV18 E6/E7 and HPV16 E6/E7 mRNA; HPV16 E6 mRNA was increased (*P < 0.05). **(F)** Decreased p53 protein expression (*P < 0.05).

To investigate the function of miR-187 in CC, we transiently transfected 40 nM miR-187 mimics into HeLa and CaSki co-cultures for 48 h and measured HPV18 E6/E7 and HPV16 E6/E7 mRNA expression. The mimics increased miR-187 expression in the cells (Figure 4A). The relative expression of HPV16 E6 mRNA was decreased (Figure 4B), and there was a corresponding increase in p53 protein expression (Figure 4C). Conversely, 48-h transient transfection with 40 nM miR-187 inhibitor markedly increased the relative expression of HPV16 E6 mRNA and decreased p53 protein expression in the co-cultures (Figure 4D–4F).

### MiR-187 overexpression inhibits CaSki cell proliferation, migration, and invasion, and promotes CaSki cell apoptosis

**Figure 5:**
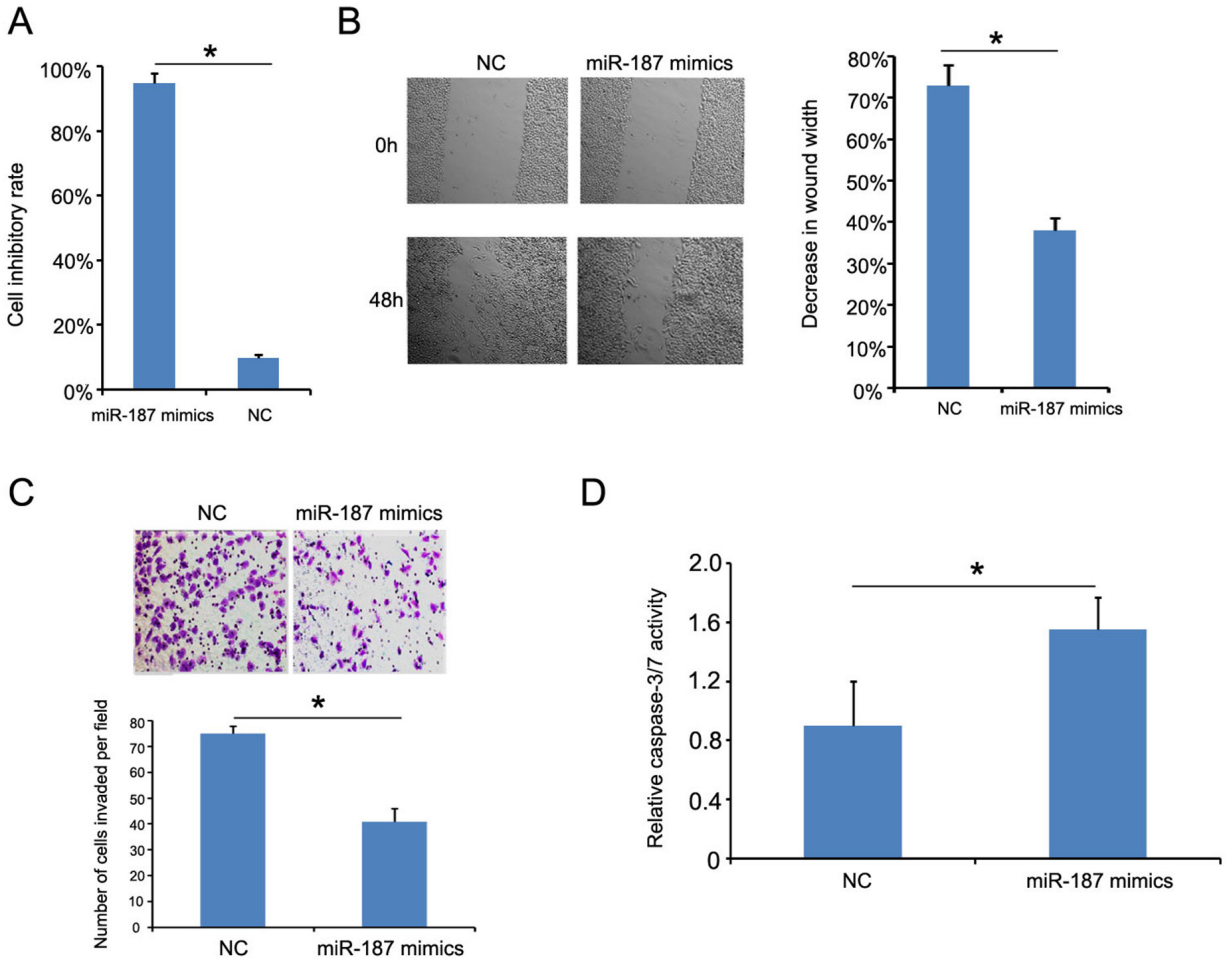
miR-187 overexpression inhibits CaSki cell proliferation, migration, and invasion, and promotes CaSki cell apoptosis **(A)** CCK-8 detection of miR-187 overexpression inhibition of CaSki cell proliferation (*P < 0.05). **(B)** Wound healing assay of CaSki cells transfected with 40 nM miR-187 mimic or NC for 48 h. Bars represent the average percentage of wound healing ± SD (*P < 0.05). **(C)** Transwell chamber measurement of CaSki cell invasive ability. Photos show representative fields of invasive cells on the membrane at ×200 magnification. Bar graphs depict the average number of cells per field on the underside of the membrane ± SD (*P < 0.05). **(D)** miR-187 overexpression increased caspase-3/-7 activity in CaSki cells compared with miR-187 NC–transfected cells. Bars represent the average percentage of caspase-3/-7 ± SD (*P < 0.05).

Cell Counting Kit-8 (CCK-8) showed that the inhibitory rate of cells growth treated with miR-187 mimics was inhibited compared with cells transfected with negative control (NC) mimics (Figure 5A). The wound healing assay was conducted to confirm the role of miR-187 in CC progress, and showed that CaSki cells overexpressing miR-187 were less efficient at closing an artificial wound compared to CaSki cells expressing miR-NC (CaSki, 48 h, Figure 5B). The Transwell assay was performed to determine whether miR-187 affects CaSki cell invasive capacity, and showed that miR-187 significantly decreased the invasive potential of CaSki cells (Figure 5C). Consistently, elevated caspase-3/-7 activity was also observed in the miR-187 mimics group (Figure 5D). Taken together, these results suggest that miR-187 over-expression inhibits CC tumor growth *in vitro*.

### Subcutaneous tumor transplantation

**Figure 6:**
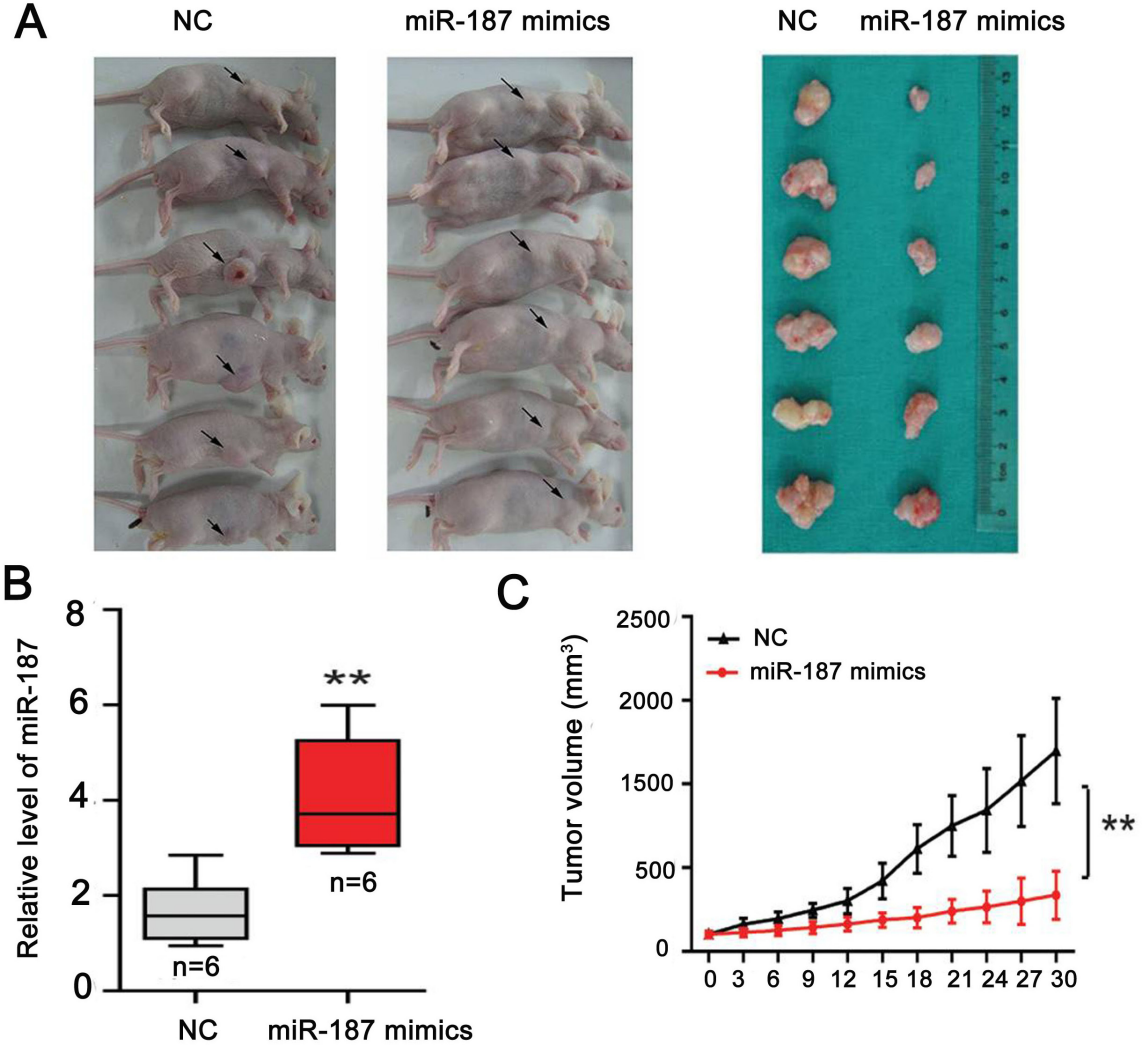
miR-187 overexpression inhibits tumor growth of CC *in vivo* **(A)** Subcutaneous tumor transplantation experiment. **(B)** Detection of miR-187 levels in tumor tissues following treatment with miR-187 mimics or NC (**P < 0.01). **(C)** The tumor mass in the miR-187 mimics group was lower than that in the NC group (**P < 0.01).

We performed a subcutaneous tumor transplantation experiment to confirm the role of miR-187 in tumor growth (Figure 6A). miR-187 mimics increased miR-187 levels in the tumor microenvironment and inhibited CaSki cell growth in nude mice (Figure 6A, 6B). Excision and measurement of the tumors revealed lower tumor mass in the miR-187 mimics group than in the NC group (Figure 6C). The results suggest that miR-187 overexpression inhibits CC tumor growth *in vivo*.

### Low miR-187 expression promotes CaSki cell proliferation, migration, and invasion, and inhibits CaSki cell apoptosis

**Figure 7:**
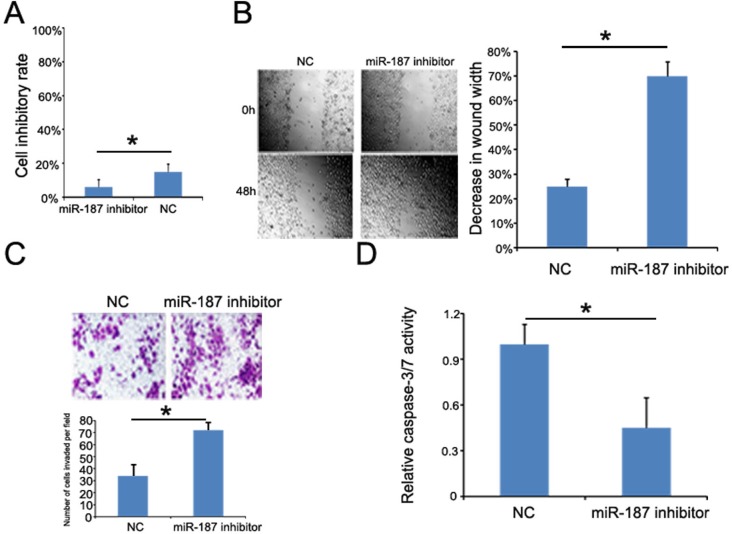
miR-187 knockdown promotes CaSki cell proliferation, migration, and invasion, and inhibits CaSki cell apoptosis **(A)** CCK-8 detection of CaSki cell proliferation promoted by low miR-187 expression (*P < 0.05). **(B)** Wound healing assay of CaSki cells transfected with 40 nM miR-187 inhibitor or NC for 48 h. Bars represent the average percentage of wound healing ± SD (*P < 0.05). **(C)** Transwell chamber determination of CaSki cell invasive ability. Photos show representative fields of invasive cells on the membrane at ×200 magnification. Bar graphs represent the average number of cells per field on the underside of the membrane ± SD (*P < 0.05). **(D)** miR-187 inhibition decreased the caspase-3/-7 activity in CaSki cells. Bars represent the average percentage of caspase-3/-7 ± SD (*P < 0.05).

We transfected CaSki cells with miR-187 inhibitor or miR-187 inhibitor control to demonstrate whether low miR-187 expression contributes to tumor progression. As expected, miR-187 suppression significantly promoted CaSki cell proliferation (Figure 7A), migration (Figure 7B), and invasion (Figure 7C). Furthermore, starvation-induced CaSki cell apoptosis was inhibited (Figure 7C). Taken together, these observations indicate that low miR-187 expression promotes CC tumor progression by negatively controlling these cellular phenotypes.

### HPV16 E6 is a direct target of miR-187

**Figure 8:**
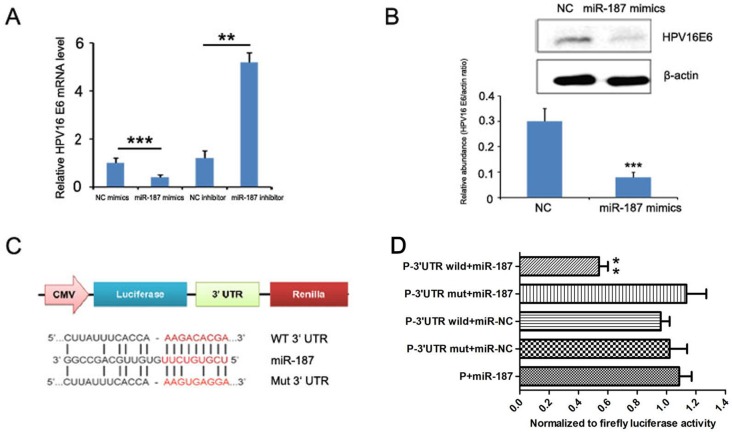
HPV16 E6 is a direct target of miR-187 **(A)** HPV16 E6 mRNA levels detected in the presence of miR-187 mimics or miR-187 inhibitor (**P < 0.01, ***P <0.001). **(B)** Western blot detection of HPV16 E6 protein levels in CaSki cells in the presence of miR-187 mimics (***P <0.001). **(C)** Bioinformatics prediction of the miR-187 binding sites of the HPV16 E6 3′ UTR. The mutated HPV16 E6 3′ UTR is also shown. **(D)** Luciferase activity of CHO cells co-transfected with miR-187 expression vector or empty vector and HPV16 E6 3′ UTR reporter plasmid or its mutant form for 48 h. Data shown are the means ± SD of three replicates (**P < 0.01).

We investigated the potential miR-187 target gene HPV16 E6 to explore the mechanism by which miR-187 affects the biological functions of CaSki cells. To verify this prediction, we detected the expression of HPV16 E6 mRNA in CaSki cells in the presence of miR-187 mimics or miR-187 inhibitor. MiR-187 mimics significantly reduced HPV16 E6 mRNA expression, and miR-187 inhibitor increased it (Figure 8A). Western blotting demonstrated that miR-187 decreased HPV16 E6 protein levels in CaSki cells (Figure 8B), which supported our hypothesis.

The dual-luciferase reporter assay was used to verify whether HPV16 E6 is a direct target of miR-187, then, a human HPV16 E6 3′UTR fragment containing the wild-type or mutant miR-187-binding site was inserted downstream of the luciferase open reading frame (Figure 8C). The reporter vector carrying the partial sequence of the HPV16 E6 3′ UTR was cotransfected with miR-187 mimic in Chinese hamster ovary (CHO) cells. Compared with the negative control a significant decline of luciferase activity was observed with miR-187 transfection (Figure 8D, P< 0.01). Moreover, the relative luciferase activity of the reporter containing wild-type HPV16 E6 3′-UTR was markedly decreased upon miR-187 co-transfection (Figure 8D, P< 0.01), whereas the luciferase activity of the reporter containing the mutant binding site was unaffected. These results strongly suggest that HPV16 E6 is a direct target of miR-187 in CHO cells.

## DISCUSSION

There have been few advances in CC treatment over the past decade. The discovery of miRNAs provides novel insight into the molecular mechanisms and treatments for cancer. The roles of miRNAs in cancer have attracted increased attention. In this study, microarray analysis detected miR-187 downregulation in CC tissues; subsequently, we measured miR-187 expression in CC tissues and cell lines, and found that miR-187 was significantly downregulated as compared with miR-187 expression in the corresponding normal controls. The biological functions of miR-187 in CC were investigated using gain- and loss-of-function studies. MiR-187 downregulation predicts poor prognosis in clear cell renal cell carcinoma, demonstrating that miR-187 downregulation is associated with higher tumor grade and stage and plays a tumor-suppressive role [17]. Similarly, we discovered that miR-187 expression levels are associated with pathological grade, clinical stage, nodal metastasis, and larger tumor size via correlation analysis of the clinicopathological characteristics of CC. Furthermore, we found that the rates of OS and relapse-free survival of the high miR-187 expression group were higher than that of the low miR-187 expression group. An increasing amount of evidence has validated the use of down-regulated and up-regulated miR-187 as a tumor suppressor gene or oncogene, respectively, and miR-187 is closely related with tumor cell proliferation and apoptosis. Ectopic expression of miR-187 has been reported in nasopharyngeal [16], renal [17], pancreatic [18], prostate [19], thyroid [20], gastric [21], and esophageal cancer [22] and in neuroblastoma [23]. These findings suggested the functions of miR-187 expression patterns may be tissue-specific. As we all know, HPV16, HPV18, HPV31, and HPV45 pose a high risk for CC; the most common cancer subtypes are HPV16 and HPV18, where HPV16 is most closely related with cervical squamous cell carcinoma and HPV18 is most closely related with cervical adenocarcinoma [24]. Therefore, we focused on the role of miR-187 in HPV16+ CaSki cell and HPV18+ HeLa cell over other HPV subtypes or HPV-negative cells. Most excitingly, we demonstrated that miR-187 acts as a tumor suppressor by targeting HPV 16E6 in CaSki cell proliferation and invasive capacity, and apoptosis. In addition to the *in vitro* experiment, we validated our findings in nude mice, where the tumors formed in nude mice treated with miR-187 mimics were significantly smaller than that of the control group. However, further research is still needed to explore whether or how miR-187 can directly target HPV16 E6.

In order to explore the mechanisms of miR-187, we identified HPV16 E6 as a putative miR-187 target gene. CC has the most severe incidence and mortality rates [2], and HPV genotype 16 (HPV16) is the most frequently detected in CC, being reported in almost 60% of CC cases worldwide [25]. HPV16 E6, known as an important early gene in CC progression, is an indispensable oncoprotein of CC incidence, and maintains the malignant phenotype. The normal wild-type P53 gene is a core member of cell cycle regulation, controlling cellular processes such as gene transcription, DNA synthesis, DNA repair, cell cycle arrest, cell senescence, and apoptosis [26]. One mechanism through which the HPV16 E6 gene influences CC development and curative effect is inactivating the tumor suppressor gene P53 in the host cell through its E6AP (E6 associated protein), causing the loss of control of normal cell regulation, unlimited proliferation, and malignant transformation [27]. Therefore, we selected HPV 16E6 for further study reasonably. Indeed, we confirmed that HPV 16E6 mRNA and protein were regulated by the high or low expression of miR-187, as showed by Real-time-PCR and Western blotting, respectively. Also we proved that HPV 16E6 is a direct target of miR-187 by dual luciferase reporter gene assay. However, HPV18 E6 and E7 mRNA expression were unchanged, indicating no association between miR-187 and HPV18 E6/E7. At the same time, miR-187 transfection had no effect on HPV16 E7, suggesting that miR-187 cannot activate HPV16 E7 in CC or HPV16 E7 might not contain high free energy binding sites of miR-187; perhaps other mechanisms not found in this research are present.

miRNAs effect changes mainly by altering target gene expression. DAB2 [14] and matrix metalloproteinase (MMP) [28] are target genes of miR-187. Sirotkin et al. [29] found that, in human ovarian granulosa cells, low miRNA expression reduces the apoptosis-related protein BAX and the expression of proliferating cell nuclear antigen (PCNA). In a miRNA–mRNA direct-interaction CLASH (crosslinking, ligation, and sequencing of hybrids) experiment, Helwak et al. [30] found that TUBG1, MAD2L2, and STOML2 were target genes of miR-187. MAD2L2 is involved in mitosis checkpoint control; its expression is upregulated in colon cancer tissue as compared with the adjacent normal tissue, and there is excessive MAD2L2 expression in patients with colorectal cancer with poor prognosis [31]. STOML2 is a stomatin superfamily member whose expression has been confirmed in a wide variety of tumor tissue [32–35]. To explore the mechanisms of miR-187 in CC, we identified HPV16 E6 as a putative miR-187 target gene, and selected it for further study. Based on the results, we discovered a new target gene HPV 16E6 of miR-187, which is an interesting and important topic.

In summary, the present study describes a novel link between miR-187 and HPV16 E6 in CC. The miR-187/HPV16 E6 axis provides novel insight into CC pathogenesis, and it might represent a potential therapeutic target in CC.

## MATERIALS AND METHODS

### Patients and tissue specimens

A total 209 cervical tissue samples, including 82 carcinoma tissues and matched 82 normal tissues (5 cm distal from the tumor) and 45 cervical squamous intraepithelial lesion samples (control) were collected from Fuda Cancer Hospital, Jinan University School of Medicine, Fuda Cancer Institute, and Peking University between January 2008 and February 2014, and the patients’ clinicopathological data were collected. No patient had received neoadjuvant chemotherapy preoperatively. Postoperative hematoxylin–eosin staining verified that all patients had CC. All fresh CC tissues were sampled immediately after surgical removal and immediately frozen in liquid nitrogen for further use within 10 min. The follow-up time was calculated from the date of surgery to the date of death or the last known follow-up. All patients provided informed consent and hospital ethics committee approved the study. Two senior pathologists made all histological diagnoses.

### Cell culture and transfection

HeLa and CaSki cell lines purchased from American Type Culture Collection (Manassas, VA, USA) and the cell bank of the Chinese Academy of Science (Shanghai, China), respectively, were cultured at 37°C in 5% CO_2_ in a humidified incubator in Dulbecco's modified Eagle's medium and RPMI 1640 medium, respectively. All cells were authenticated by short tandem repeat profiling before receipt and were propagated for less than 6 months after resuscitation.

miRNAs were transfected at a working concentration of 100 nmol/L using Lipofectamine 2000 reagent (Invitrogen, Carlsbad, CA, USA). miR-187 mimics, a nonspecific mimic control, miR-187 inhibitor, and a nonspecific inhibitor control were all purchased from GenePharma (Shanghai, China). Protein and RNA samples were extracted from subconfluent cells during the exponential growth phase.

### Total RNA extraction and real-time PCR

The qRT-PCR for miRNA and mRNA was performed after the concentration of total RNA extracted with TRIzol (Invitrogen) was calculated by measuring the absorbance at A260/280. For miRNA quantification, each RT reaction was performed in a final volume of 10 μL consisting of 0.5 μg total RNA, 2.0 μL 5× RT buffer containing dNTPs (Takara Bio, Otsu, Japan), 0.2 μL 10 μmol/L stem-loop RT primer (Invitrogen), 0.2 μL RNase inhibitor protein (Takara Bio), and 0.8 μL reverse transcriptase (Takara Bio), and incubated at 42°C for 60 min and at 85°C for 5 min. Real-time PCR was performed in triplicate using an Applied Biosystems 7500H system (Foster City, CA, USA) using SYBR Premix Ex Taq (Takara Bio). Cycling conditions were 1 cycle at 95°C for 30 s and 40 cycles at 95°C for 5 s and 60°C for 30 s. Total complementary DNA (cDNA) was synthesized using a TaKaRa RT kit (Takara Bio). Real-time PCR was performed using SYBR Premix Ex Taq (Takara Bio). Table 2 lists the primers used and their sequences. Relative expression was determined by normalizing the expression of each threshold cycle (Ct) value to that of glyceraldehyde-3-phosphate dehydrogenase (GAPDH) Ct value, and data were analyzed according to the comparative Ct method (2^−ΔΔCt^).

**Table 2:** Primer sequences

Primer		Sequence (5′–3′)	Product
GAPDH	Forward	CAGGGCTGCTTTTAACTCTGGTAA	101 bp
	Reverse	GGGTGGAATCATATTGGAACATGT	
HPV18 E6	Forward	CCAGAAACCGTTGAATCC	126 bp
	Reverse	AGTCGTTCCTGTCTGTCTC	
HPV18 E7	Forward	CACGAGCAATTAAGCGACT	140 bp
	Reverse	GCTCAATTCTGGCTTCAC	
HPV16 E6	Forward	GAGCGACCCAGAAAGTTACCAC	107 bp
	Reverse	ACCTCACGTCGCAGTAACTGTTG	
HPV16 E7	Forward	GATCTCTACTGTTATGAGCAA	140 bp
	Reverse	AACCGAAGCGTAGAGTCACA	
miR-187	RT stem-loop primer	GTCGTATCCAGTGCAGGGTCCGCGCACTGGATACGACCCGGCTGC	64 bp
	Forward	TCGTGGGTCGTGTCTTGTGTTGC	
	Reverse	GCAGGGTCCGAGGTATTC	
U6	RT stem-loop primer	GCTTCGGCAGCCACATATACTAAAAT	101 bp
	Forward	CGCTTCACGAATTTGCGTGTCAT	
	Reverse	GGGTGGAATCATATTGGAACATG	

### Cell proliferation assay

For each treatment, 5000 cells per well were seeded in a 96-well culture plate and grown at 37°C overnight. Cell viability was quantified using CCK-8 (Dojindo, Kumamoto, Japan). Briefly, on the day the growth rate of the treated cells was measured, 100 μL spent medium was replaced with an equal volume of fresh medium containing 10% CCK-8 reagent, and then the cells were incubated at 37°C for another 1–4 h before the absorbance was determined at 450 nm using a microplate reader.

### Cell migration and invasion assay

An *in vitro* wound healing assay was performed to assess cell motility. CaSki cells (4 × 10^5^/well) were seeded in 12-well plates and transfected with miR-187 mimics. When the cultures were almost 90% confluent, the monolayers were scratched with a 10-μL sterile plastic pipette tip and the cellular debris was washed away with phosphate-buffered saline. The cells were cultured again in Opti-MEM at 37°C in a humidified incubator with 5% CO_2_. The plates were photographed and wound healing was measured at 0 h, 24 h, and 48 h, and data from six assays per experiment were summarized.

The invasion assay was performed in Transwell chambers with membranes coated with Cultrex Basement Membrane Extract without Phenol Red (R&D Systems, Minneapolis, MN, USA). CC cells (5 × 10^4^) were placed in the top chamber of each insert, which had been coated with 100 μL 2 mg/mL growth factor–reduced Matrigel (BD Bioscience, San Jose, CA, USA), and 700 μL RPMI 1640 medium with 5% fetal bovine serum was added to the bottom chamber. After 48-h incubation, the chambers were disassembled, the non-invaded cells that remained in the top chamber were removed, and the membranes were stained with 2% crystal violet solution for 30 min and placed on a glass slide. The cells in each chamber were photographed and counted under ×100 magnification. All invasion assays were performed in triplicate in at least three independent experiments.

### Apoptosis assay

For the caspase-3/-7 activity assay, CC cells (10,000 /well) were seeded on 96-well plates. After 48-h starvation, cell numbers and caspase-3/-7 activity in the same sample were monitored using CellTiter-Blue (Promega, Madison, WI, USA, #G8081) and Apo-ONE Caspase-3/7 assay (Promega, #G7790), respectively. Caspase-3/-7 activity was calculated as the ratio of Apo-ONE/CellTiter-Blue signals. The measurement was performed in triplicate.

### Western blotting

Cell lysates were prepared using radioimmunoprecipitation assay lysis buffer in the presence of protease inhibitors. Protein concentrations were determined using a BCA Protein Assay Kit (Thermo Fisher Scientific, Waltham, MA, USA). Primary antibodies against p53 (DO-1) and β-actin were purchased from Abcam. Rabbit anti-human HPV16 E6 antibody (BS1719-R, 1:400) was purchased from BIOSS. Secondary antibody against goat anti-rabbit immunoglobulin G horseradish peroxidase conjugate was purchased from AJ. Proteins (30 μg) were separated on 10% sodium dodecyl sulfate–polyacrylamide gel and transferred to a nitrocellulose membrane (Bio-Rad, Hercules, CA, USA). Then, the membrane was blocked with 5% non-fat milk for 2 h and incubated with primary antibodies. Proteins were detected using enhanced chemiluminescence reagents (Thermo Fisher Scientific). For immunoreactivity, the membrane was rinsed with TBS, incubated overnight at 4°C with a primary antibody, and then washed and incubated for 1 h with secondary antibody. Immunoreactivity was detected using an enhanced chemiluminescence substrate (Thermo Fisher Scientific). Lab Works image acquisition and analysis software (UVP, LLC) was used to quantify band intensities.

### Tumorigenesis in nude mice

Xenograft tumors were generated by subcutaneous injection of 4 × 10^6^ cells into the hind limbs of 4–6-week-old BALB/C athymic nude (nu/nu) mice obtained from the animal center of East China Normal University, Shanghai, China. The mice were housed and maintained under specific pathogen–free conditions and used in accordance with institutional guidelines and the Use Committee for Animal Care approved the study protocol. Tumor size was measured using a slide caliper every 3 days; tumor volume was determined as follows: 0.44 × A × B^2^ (A, tumor base diameter in one direction; B, the corresponding perpendicular value). When the average tumor size was 100 mm^3^, the mice were randomly separated into two groups subcutaneously injected with miR-187 mimics or miR-187 NC at different sites. The injections were performed twice a week. The mice were sacrificed 30 days after the initial injection, and the tumors were excised.

### Luciferase reporter assay

The pmirGLO dual-luciferase miRNA target expression vector (pmirGLO vector) containing both the firefly luciferase and Renilla luciferase genes was purchased from Promega. Human HPV16 E6 3′ UTR containing the predicted miR-187 binding site was inserted into the 3′ UTR downstream of the firefly luciferase gene of the pmirGLO vector (pmir-GLO-UTR). A site-directed mutagenesis kit (Beyotime, Jiangsu, China) was used to construct the mutant miR-187-binding site vector (pmir-GLO-mUTR) according to the manufacturer's protocol. CHO cell co-transfection with miRNA mimics (40 nM) and reporter vectors (0.2 μg/mL) were performed using Lipofectamine 2000 (Invitrogen). After 48 h, the cells were harvested and lysed, and luciferase activity was measured using the Dual-Luciferase Reporter Assay System (Promega). Renilla luciferase was used for normalization. The experiments were performed independently in triplicate.

### Statistical analyses

Data are expressed as the means ± SD of at least three independent experiments. All statistical analyses were performed using SPSS 16.0 software; graphical representations were performed using GraphPad Prism 5 (San Diego, CA, USA). The OS rate was calculated using the Kaplan–Meier method, and the difference in survival curves was evaluated using the log-rank test. The Student *t*-test was used to analyze differences between two groups, and one-way analysis of variance was used to determine the significance of differences among multiple groups. P-values < 0.05 were considered statistically significant.

## Abbreviations

2-ΔΔCt: comparative Ct method
3′ UTR: 3′ untranslated region
CC: cervical cancer
cDNA: complementary DNA
CLASH: crosslinking, ligation, and sequencing of hybrids
Ct: threshold cycle
DAB2: disabled homolog-2
GAPDH: glyceraldehyde-3- phosphate dehydrogenase
HPV: human papillomavirus
HSIL: high-grade squamous intraepithelial lesion
LSIL: low-grade squamous intraepithelial lesion
miRNA: microRNA
MMP: matrix metalloproteinase
OS: overall survival
PCNA: proliferating cell nuclear antigen
qPCR: quantitative PCR
RT-qPCR: reverse transcription–qPCR
